# *QuickStats:* Age-Adjusted Rates* of Alcohol-Induced Deaths,^†^ by Urban-Rural Status^§^ — United States, 2000–2020

**DOI:** 10.15585/mmwr.mm7144a5

**Published:** 2022-11-04

**Figure:**
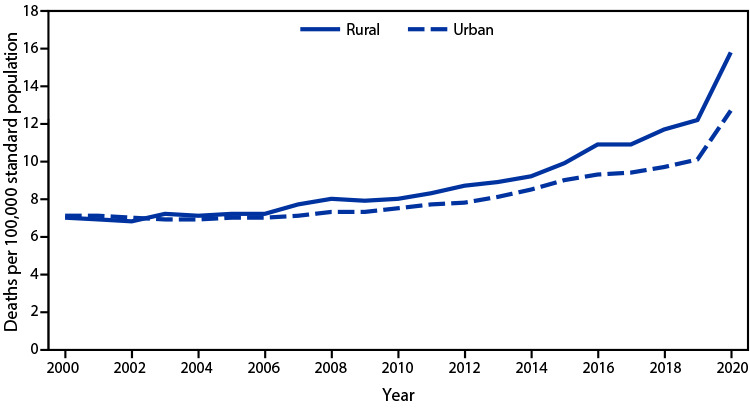
The age-adjusted rate of alcohol-induced deaths in 2020 was 13.1 per 100,000 standard population. From 2000 to 2020, the rate increased in both urban and rural counties: from 7.1 to 12.7 in urban counties and from 7.0 to 15.8 in rural counties. From 2019 to 2020, the rate increased by 26% for urban counties and 30% for rural counties, which was the largest increase for both urban and rural counties during the 2000–2020 period.  Rates were similar between rural and urban counties from 2000 to 2004, but from 2005 to 2020 rates were higher in rural counties than in urban counties. During 2005–2020, rural rates increased at a greater pace than did urban rates. By 2020, the rate in rural counties was 24% higher than in urban counties.

**Source:** National Vital Statistics System, Mortality Data. https://www.cdc.gov/nchs/nvss/deaths.htm

